# Isolation and identification of bacteria from blood within 12 h using standard laboratory equipment

**DOI:** 10.1038/s41598-025-09024-9

**Published:** 2025-07-09

**Authors:** Maria Henar Marino Miguélez, Alan Huguenin-Dumittan, Mohammad Osaid, Wouter van der Wijngaart

**Affiliations:** 1 Micro and Nanosystems, KTH Royal Institute of Technology, Stockholm, Sweden; 2 School of Life Sciences, École Polytechnique Fédérale de Lausanne, Lausanne, Switzerland

**Keywords:** Sepsis, Bacteria, Bacteremia, Bloodstream Infections, Diagnostics, Clinical microbiology, Bacterial infection, Preclinical research

## Abstract

Sepsis has an incidence of 50 million cases per year and represents a significant cause of morbidity and mortality worldwide. Current diagnostic methods rely on blood drawn directly into blood culture media in hemoculture bottles, followed by culturing, often taking days to yield results and failing to meet urgent clinical needs. We present here a protocol for isolating and identifying bacteria from blood within 12 h after sampling, bypassing prior hemocultures. Starting from blood added into blood culture media, according to standard hospital sampling practice, we isolated up to 85% of bacteria at clinically-relevant concentrations in less than 15 min using an optimized centrifugation protocol. Subsequent overnight culture of the isolated bacteria on chromogenic agar plates enabled species identification of five of the most prevalent sepsis-causing bacteria. The rapidity and simplicity of the protocol may accelerate the diagnostic pipeline for sepsis patients. Moreover, the use of standard laboratory equipment may enable direct translation to clinical praxis and concatenation with downstream assays for antibiotic susceptibility testing.

## Introduction

Sepsis has an incidence of around 50 million cases annually, making it a significant healthcare burden. With a mortality rate between 25% and 40%^1^, sepsis accounts for approximately 1 in 5 deaths globally or approximately 11 million deaths per year^2^. It is estimated that more than half of septic patients in the intensive care unit (ICU) present bacteria in the bloodstream, also called bacteremia^3^. Confirming the presence of bacteria or other pathogens in the blood often provides decisive evidence of bloodstream infection in patients. Therefore, detecting and identifying pathogens in blood is an essential step toward successfully treating these infections^4^. Microbiological studies for the detection of pathogens in blood continue to rely mostly on conventional culture-based systems (hemocultures), which remain the gold standard^5^. To perform a hemoculture, a sample of blood is obtained from the patient and transferred to a sterile flask containing a specialized blood culture medium (BCM) (most commonly trypticase soy broth) designed to support the growth and proliferation of microorganisms. The container is then incubated in a laboratory, usually at body temperature, to allow the growth of bacteria or fungi present in the blood. Blood culture positivity is usually detected by monitoring $\hbox {CO}_2$ production from growing microorganisms, which decreases pH and triggers detectable changes in color, fluorescence or redox signals^6^. This approach takes between 12 and 36 h to yield results for most bacterial pathogens, even extending to several days in the case of some fastidious bacteria, anaerobes and fungi^7^.

Conventionally, species identification (species ID) of the causative pathogens has been based on biochemical tests, which are lengthy and time-consuming^8^. However, in the past decade, several new technologies have emerged to speed up the identification of microbial pathogens. These include protein-based identification, such as matrix-assisted laser desorption ionization-time of flight mass spectrometry (MALDI-TOF MS)^9,10^, and nucleic acid-based identification, such as polymerase chain reaction (PCR)^11,12^, loop-mediated isothermal amplification (LAMP) of molecular targets^13,14^, or peptide nucleic acid fluorescence in-situ hybridization (PNA-FISH) assays^15,16^. These new diagnostic platforms improve diagnosis speed but still require hemoculturing until positivity, which remains the rate-limiting step in the diagnosis^17^. Alternatively, a simple approach for species identification is chromogenic agar-based identification, which enables the differentiation of bacterial species based on the colorimetric changes induced by specific enzymatic activity, allowing for rapid preliminary identification of the causative pathogen^18–21^.

To reduce the overall turnaround time (TAT) of sample processing and accelerate the diagnostic pipeline, different methods have been proposed to directly isolate and identify bacteria from blood, including centrifugal sedimentation^22–24^, dean flow focusing^25^, hydrodynamic focusing^26^, dielectrophoresis^27,28^, chemical capture^29–31^ and acoustic separation^32^. The main challenge lies in the low number of bacteria, 1–100 colony-forming units (CFU) per mL^33^, relative to the high number of blood cells, typically 4–6 billion per mL, in clinical samples of bloodstream infections. However, most of these direct isolation methods necessitate highly specialized equipment or have unsatisfying limits of detection. Moreover, most methods separate bacteria directly from whole blood, which differs from the current praxis where blood is drawn directly into hemoculture bottles^34^.

With the aim to prescribe more timely an effective antibiotic treatment and better patient outcomes, we investigated the rapid isolation and identification of bacteria directly from blood drawn into hemoculture bottles, relying solely on techniques widely available in hospital laboratory settings.

## Results

### Overall workflow

**Fig. 1:**
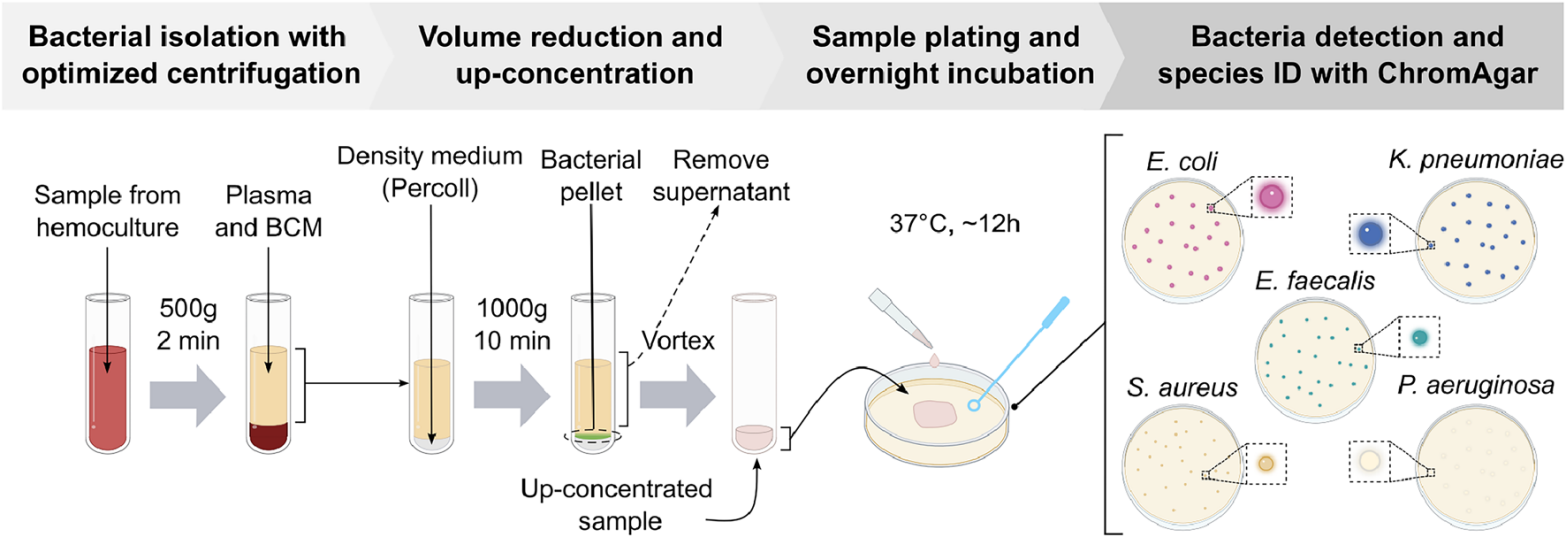
The protocol to isolate, detect, and identify bacteria from hemocultures concatenates four assay steps: bacterial isolation with optimized centrifugation parameters; volume reduction and up-concentration with high-density medium (Percoll); sample plating and overnight ($\ge$ 12 h) incubation; and bacteria detection and species identification with chromogenic agar plates for *E. coli*, *K. pneumoniae*, *S. aureus*, *E. faecalis* and *P. aeruginosa*.

We developed a protocol to efficiently isolate, detect, and identify bacteria directly from hemocultures, concatenating four different steps: bacteria isolation by centrifugation with optimized parameters, volume reduction and up-concentration, sample plating and overnight incubation on chromogenic agar, and bacteria detection and species ID (Fig. 1). All experiments started with healthy human EDTA donor blood, added to the media obtained from a standard aerobic hemocultucure bottle (BD BACTEC Plus Aerobic medium, BD, USA) and spiked with bacteria from five of the most prevalent species in septic patients’ blood: *Escherichia coli*, *Klebsiella pneumoniae*, *Staphylococcus aureus*, *Enterococcus faecalis* and *Pseudomonas aeruginosa*. These species were chosen because they cumulatively appear in 50.6% of the hospital-acquired bloodstream infections (BSIs) worldwide^35^, 48.4% of ICU patients with sepsis in Japan^36^, 77.3% of ICU patients with sepsis and detected bacteremia in Japan^3^, and more than 49.3% of detected bacteremias in Lausanne’s University Hospital (Switzerland)^6^ (SI Table 1).

**Fig. 2:**
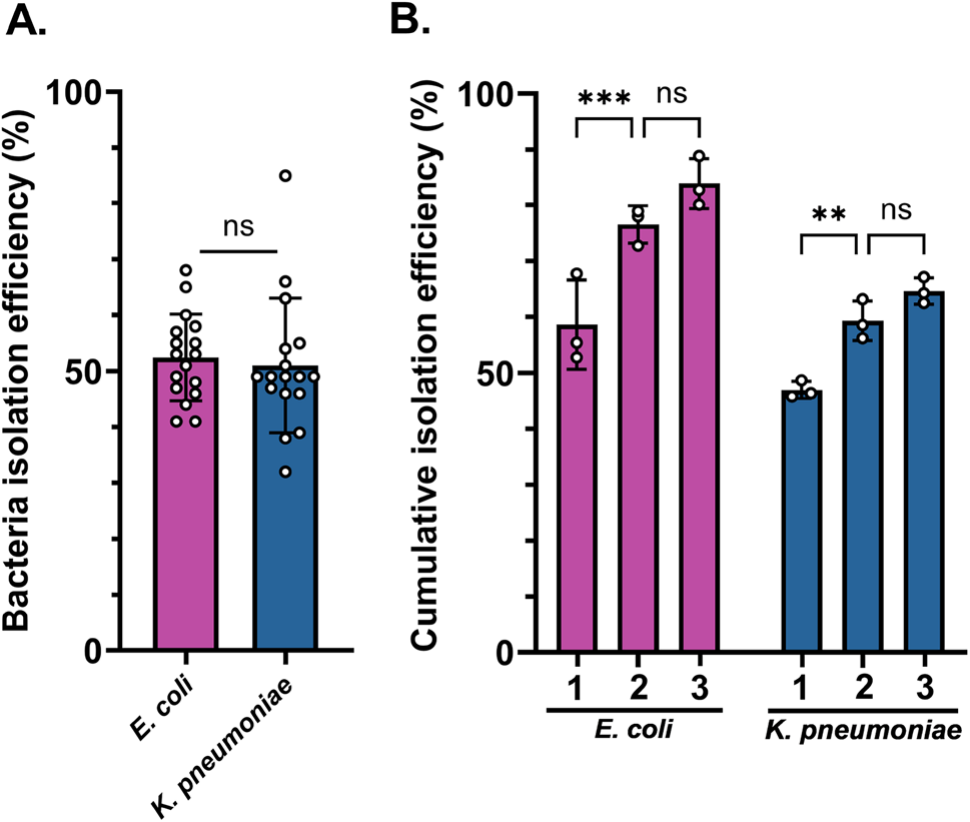
Isolation efficiency of *E. coli * and *K. pneumoniae.* (**A**) *E. coli* and *K. pneumoniae* isolation efficiency after optimized centrifugation (500*g* for 2 min). Statistical test: student’s unpaired t-test. (**B**) *E. coli* and *K. pneumoniae* cumulative bacteria isolation efficiency after 1, 2, and 3 iterations of the separation spin. Statistical test: Two-Way ANOVA. Mean and standard deviation are shown; ns indicates significance level with *p*-value $\ge$ 0.05. **Indicates significance level with *p*-value < 0.01 and *** indicates significance level with *p*-value < 0.001.

### Bacterial isolation from hemocultures

We optimized centrifugation parameters for separating bacteria from hemocultures in 15 mL tubes containing 6 mL of cultured blood by adjusting centrifugation time and relative centrifugal force (RCF). We experimentally tested several combinations of centrifugal parameters to maximize the *E. coli* isolation and red blood cell removal efficiency, while minimizing the centrifugation time. The optimized centrifugation time and RCF were determined to be 500*g* and 2 min, respectively (see SI section Optimization of the operational centrifugal parameters for isolation of bacteria from hemocultures). Following this centrifugation step, approximately 4 mL of supernatant can be extracted.

The parameter optimization was performed using blood spiked with *E. coli* at concentrations, *C*, in the range of 900–1700 CFU/mL, and subsequently validated with blood spiked with concentrations ranging between 500 and 2300 CFU/mL of *K. pneumoniae*. The bacteria isolation efficiency of *E. coli* (n = 17) and *K. pneumoniae* (n = 17) were $53 \pm 8 \%$ and $51 \pm 12 \%$, respectively. Those results do not significantly differ (*p*-value = 0.89; Fig. 2A) and the red blood cell (RBC) removal for these parameters is >99% (SI Figure 1).

We demonstrated the possibility of further increasing the bacteria isolation efficiency by resuspending the blood sediment in 4 mL of BCM, and iteratively performing the same centrifugation step up to three times. The cumulative isolation efficiency increased with each iteration. A second iteration led to a statistically significant increase of recovery to $77 \pm 3 \%$ and $59 \pm 4 \%$ for *E. coli* and *K. pneumoniae* respectively, while a third spin increased the cumulative yield to $84 \pm 5 \%$ and $65 \pm 2 \%$ (Fig. 2B). Although optional, this additional step increases the likelihood of detecting bacteria, especially in samples with low bacterial concentrations.

### Bacterial up-concentration and sample volume reduction

The 4 mL supernatant extracted after a single centrifugation step is too large to be plated in a clinical setting. To reduce the volume and up-concentrate the bacteria, we placed the supernatant on top of 200 μL density medium (Percoll, Sigma-Aldrich, USA), followed by 10 min centrifugation and subsequent removal of the bacteria-free supernatant. The density medium was added as a “cushion” to reduce centrifugal compression of the bacterial pellet. We thereafter resuspended, by vortexing, the bacterial pellet in the remaining liquid volume of approximately 0.5–0.7 mL.

**Fig. 3:**
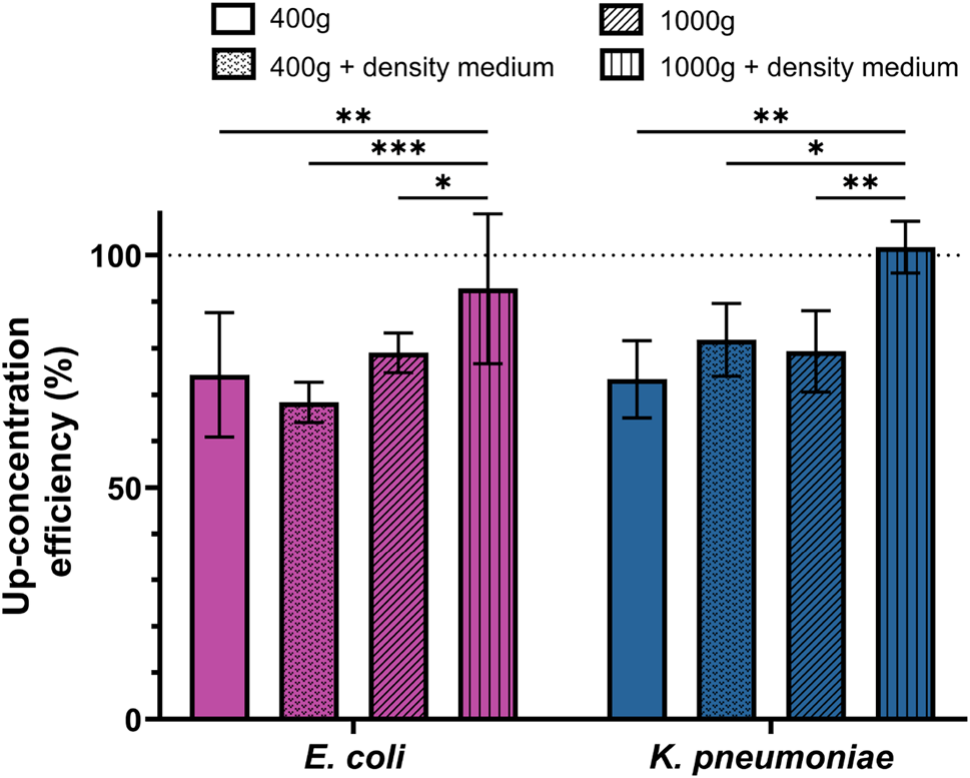
Efficiency of pelleting step (percentage of bacteria recovered after pelleting compared to the bacterial count before pelleting) for *E. coli* and *K. pneumoniae*. The pelleting parameters tested are 400g or 1000g, and presence or absence of 200 μL density medium at the bottom of the tube. Mean and standard deviation are shown. Statistical test: Two-Way ANOVA. *Indicates significance level with *p*-value < 0.05, ** indicates significance level with *p*-value < 0.01 and *** indicates significance level with *p*-value < 0.001.

We tested the efficiency of this upconcentration step alone (i.e., not accounting for the bacterial loss during the first isolation step) for *E. coli* and *K. pneumoniae* at RCF values of 400g and 1000g, with and without the presence of the density medium (Percoll). We found that recovery at 1000g on top of density medium resulted in the (near) complete recovery of the bacteria (Fig. 3), also significantly higher than with the basic setting of 400g 10min. To confirm this finding, we additionally compared the upconcentration efficiency at 1000*g* with and without density media for *S. aureus*, *E. faecalis* and *P. aeruginosa* (Figure SI 2), and showed an improved recovery for all three species.

### Species identification with chromogenic agar

**Fig. 4:**
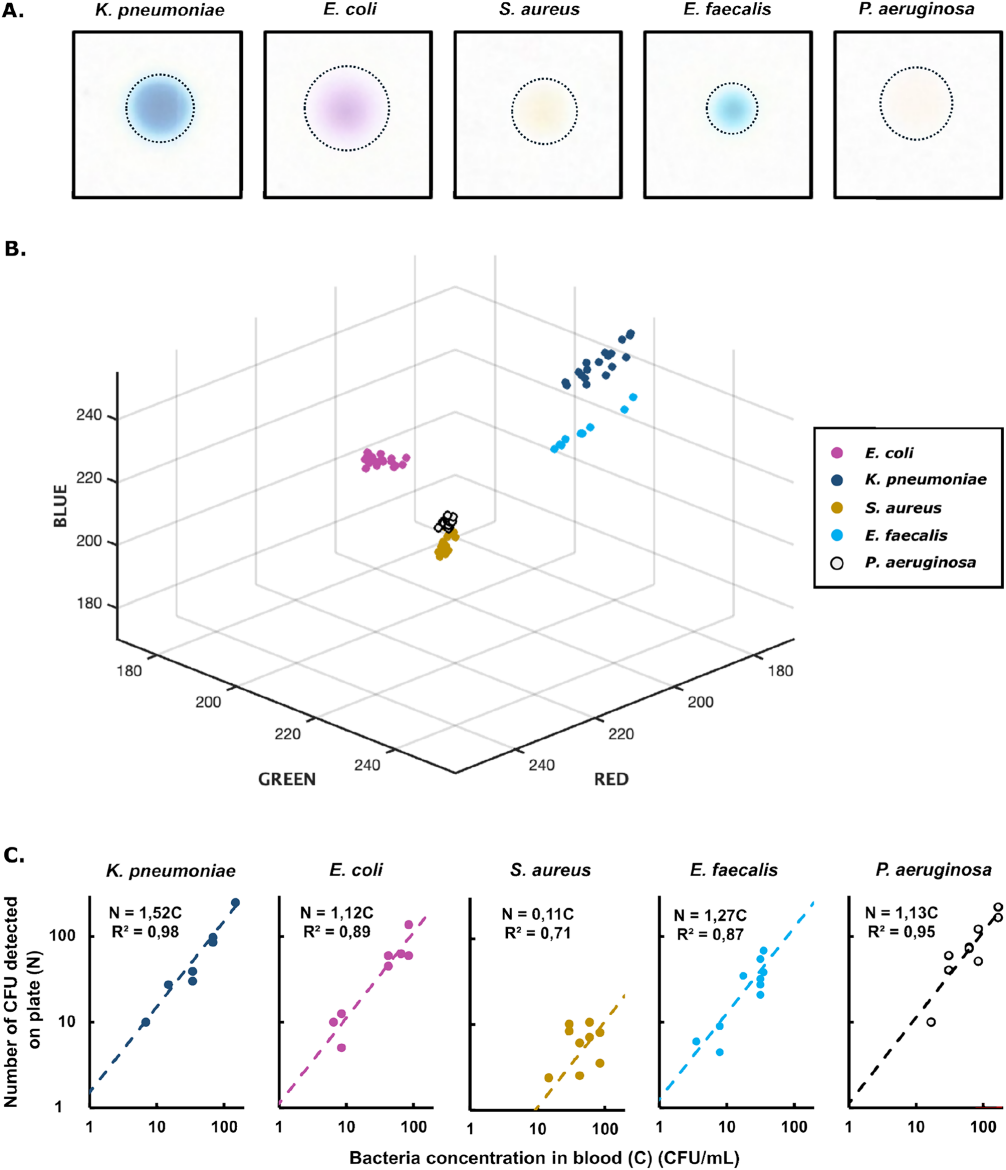
Species ID on CHROMagar and overall yield. (**A**) Photographs of single colonies of *K. pneumoniae*, *E. coli*, *S. aureus*, *E. faecalis*, and *P. aeruginosa* on chromogenic agar plates following overnight incubation at $\hbox {37}^\circ$C. The imaged colonies originate from experiments carried out at clinically relevant concentrations of $\le$150 CFU/mL and were corrected for background colour as described in SI Section “Image Analysis of ChromAgar Bacterial Colonies”. (**B**) Colony colours in the red-green-blue (RGB) colour space, where every dot is the average colour of one individual colony on a ChromAgar Orientation plate, following overnight incubation after isolation from blood. Different colours indicate different bacterial species. (**C**) Overall yield of the method, i.e., plot of the on-plate colony count, *N*, versus the bacterial concentration in the sample, *C*, for five of the most common sepsis-causing bacteria (from left to right: *K. pneumoniae*, *E. coli*, *S. aureus*, *E. faecalis* and *P. aeruginosa*). Dashed lines indicate the least square-fitting linear calibration curves $N = \eta \cdot C$.

From the up-concentrated sample, we plated 100 µL aliquots onto chromogenic agar plates (n = 2 or 3) and carefully streaked them until homogeneously distributed. After overnight incubation at $\hbox {37}^\circ$C, we determined the colony colors in RGB space using photography and automated correction for background color. Distinct colony colors on chromogenic agar allowed for accurate identification of the five tested bacterial species (Fig. 4A). The colonies from *E. coli* (n = 18), *K. pneumoniae* (n = 18) and *E. faecalis* (n = 9) cluster in clearly distinct respective zones of the RGB space, while the color of the colonies from *P. aeruginosa* (n = 21) and *S. aureus* (n = 25) show minimal overlap (Fig. 4B). For the latter, two colonies of the *S. aureus* samples were classified in the *P. aeruginosa* cluster after analysis. Overall, 89 out of 91 colony colors occupy non-overlapping regions in the color space. The different sets, though containing two overlapping points, remain uniquely identifiable in color space.

### Overall assay performance

We tested the entire protocol starting from blood samples spiked with strains of *K. pneumoniae*, *E. coli*, *S. aureus*, *E. faecalis* , and *P. aeruginosa* at clinically relevant concentrations, *C*, in the range of 10-200 CFU/mL of blood. We detected bacteria in all of the samples (Fig. 4C). The overall assay detection limit can be defined as the bacterial concentration *C* corresponding to a single colony *N* = 1. This value was estimated for each of the strains by extrapolating the linear regression curve in the plot of the on-plate colony count, *N*, versus the bacterial concentration in the sample, *C*. For *K. pneumoniae*, *E. coli*, *E. faecalis*, and *P. aeruginosa* the detection limit was approximately 1 CFU/mL, and for *S. aureus* approximately 10 CFU/mL.

## Discussion

We developed an efficient protocol to isolate and identify bacteria at clinically relevant concentrations (<100 CFU/mL) directly from hemocultures within 12 h. Its rapidity and sensitivity, and initial indications of specificity, promise to enable faster prescription of effective antibiotics compared to state-of-the-art methods, ultimately contributing to better patient outcomes. By demonstrating the protocol’s efficacy for five of the most common sepsis-causing bacterial species–accounting for more than half of bloodstream infection cases worldwide—we highlight its potential for real-world application. The estimated detection limits are approximately 1 CFU/mL for *K. pneumoniae*, *E. coli*, *E. faecalis*, and *P. aeruginosa*, and around 10 CFU/mL for *S. aureus*, addressing the sensitivity requirements for clinical diagnostics of bloodstream infections, where bacterial loads can be extremely low. Additionally, the protocol’s yield remains linear regardless of the initial bacterial concentration (Fig. 4C), underscoring its reliability. Furthermore, our protocol operates on standard laboratory equipment, ensuring compatibility with routine microbiological workflows without the need for specialized instruments. As a result, it can be seamlessly integrated into existing diagnostic pipelines in both high-income and resource-limited settings, facilitating its adoption in clinical laboratories.

In the first centrifugation step, bacterial isolation relies on the approximately 30-fold lower sedimentation velocity of bacteria compared to RBCs, due to differences in size and hydrodynamic radius^37^. In just 2 min, > 50% *E. coli* or *K. pneumoniae* can thus be separated from >99% of RBCs (SI Figure 1). This high level of erythrocyte rejection can enhance the accuracy and sensitivity of downstream diagnostic assays, as shown in previous studies^38,39^. The recovery for both *E. coli* and *K. pneumoniae* does not differ significantly (*p*-val = 0.89), which is expected as both bacterial species are bacilli of comparable shape and size, resulting in similar Stokes’ drag. By resuspending the pelleted RBC sediment and repeating the centrifugation iteratively (up to three times), an incremental increase in the cumulative recovery of organisms can be achieved, as illustrated in Fig. 2B. Each iteration corresponds to an additional centrifugation round (only the first step of the protocol). Our results show that a second round leads to a statistically significant improvement, suggesting it can be beneficial in samples with low bacterial concentrations. However, a third round does not provide a statistically significant gain over the second, with only a marginal increase in yield (6–7%) that may not justify the additional processing.

However, from a practical perspective, even though the additional hands-on time per iteration is minimal (less than 2 min), we acknowledge that in clinical settings, which are commonly understaffed, the real-world suitability of this workflow is limited unless supported by robotic automation.

Most microbiological assays start from small aliquots. This motivated our inclusion of a centrifugal bacterial up-concentration step, which purpose is to reduce the sample volume without loss of bacteria. We initially found that increasing centrifugal acceleration alone, from 400g to 1000g, did not enhance bacterial recovery. We identified a critical issue with blood component residues, which cause pellet stickiness and hinder uniform resuspension, as reported in previous studies^40,41^. We hypothesized that pelleting the particles against a high-density medium, which would act as a cushioning fluid, such as Percoll, rather than against the hard bottom of the tube, would reduce the interparticle forces in the sediment and thereby mitigate this issue. Thus, we investigated the addition of Percoll at the tube’s bottom before up-concentrating. We found, indeed, a significant improvement of bacterial recovery, from 76% (without density medim) to over 90% (at 1000g for 10 min with density medium) for both species tested. However, at 400g for 10 min with the density medium, recovery rates did not significantly differ from those without it, highlighting the importance of sufficient centrifugal forces in conjunction with the density medium for optimal bacterial recovery.

CHROMagar may function as a practical tool for low-income settings, allowing for a rapid and cost-effective early species-level identification of common sepsis pathogens (up to 56% of the pathogens^3^), without the need for advanced laboratory infrastructure, which may provide a significant diagnostic advantage. Results suggest that our protocol yields accurate bacterial identification for a significant proportion of samples, however, the limited number of samples tested in this proof-of-principle study prevents a rigorous quantification of our protocol’s specificity. Previous studies have reported the specificity of CHROMagar Orientation to be 98%^18^. In our results, colonies of *E. coli*, *K. pneumoniae* and *E. faecalis* were clearly distinguishable. The colors of *P. aeruginosa* and *S. aureus* colonies had minimal overlap (2 out of 25 *S. aureus* colonies appeared in *P. aeruginosa*’s cluster). Despite containing two overlapping points, the colony color measurements for each plate remain uniquely identifiable in color space, allowing specific species identification.

When compared to the current clinical standard for species identification (MALDI-TOF, PCR or FISH), our protocol allows for both faster detection and species ID, 12 h TAT compared to several days^4^, at the cost of a limited species coverage. MALDI-TOF, PCR or FISH rely on blood culturing, which can take 12–36 h or more to yield results^42^. Despite the availability of direct-from-positive blood culture identification methods (e.g. Sepsityper) and simplified reagent-based protocols^43–46^, due to limited access to standardized automation and lack of seamless workflow integration, many clinical laboratories continue to rely on solid media subculture of 4–24 h^47,48^,which further postpones species-specific results. In contrast, our method isolates the bacteria from the blood cells within minutes without a priori culture, before plating, allowing a sampling-to-answer TAT < 12 h. Our protocol thus allows for quicker decision-making and potentially better patient outcomes.

CHROMagar Orientation only differentiates between 10 different bacterial pathogens, which inherently limits our protocol. Therefore, the main drawback of CHROMagar is the lack of pathogen coverage, which can lead to the misidentification or failure to detect less common organisms. However, we speculate that a combination with other types of CHROMagar can help to expand the number of identifiable species. In comparison, both FISH and PCR are also limited in terms of the number of pathogens that can be detected^6,49^, whereas MALDI-TOF covers up to 1000 pathogens^6^. Moreover, in settings equipped with MALDI-TOF, this protocol can be extended by performing species ID directly from single colonies (from culture on agar plates), further enhancing its diagnostic utility.

Several rapid culture-independent techniques allow detecting bloodstream pathogens^4^, but these are expensive for most diagnostic laboratories in developing countries. In contrast, our method relies solely on standard laboratory equipment, making it an affordable and reliable alternative for bacterial identification. This is particularly important in middle- and low-income countries, as it increases global access to advanced diagnostic capabilities.

While our protocol allowed detecting all the bacteria tested in this work at clinically relevant concentrations (<100 CFU/mL), one limitation of the protocol is the higher limit of detection for *S. aureus* compared to the other species tested. This observation corroborates previous literature^22,24^ and is attributed to the ability of *S. aureus* to promote interaction between bacterial clumping factors and host proteins present in the bloodstream. This results in the formation of clusters that sediment faster during centrifugation, leading to a lower isolation efficiency^24^. While Argatroban Monohydrate has been proposed to counteract clotting^22^, its post-sampling use is ineffective due to pre-sampling clot formation. Instead, we speculate that incorporating fibrinolytic agents into the mixture before the separation spin might dissolve the clots, and thus increase the recovery of *S. aureus*. We believe that this aspect deserves further investigation in future work.

While our protocol relies on commonly available laboratory reagents and equipment, it does include the use of the density medium Percoll, which may not be universally considered a standard reagent. However, Percoll is commercially available, and we speculate that other density media with similar properties could serve as suitable alternatives.

The main limitation of this work is the lack of automation. Although the protocol requires only 2–3 min of hands-on time per sample, its implementation in high-throughput clinical settings may be impractical without automation; however, its simplicity makes it well-suited for future adaptation to robotic workflows. Another limitation of our work is that the five bacterial strains tested appear in around half of septic patients in the ICU^3,6,35,36^. There is thus a lack of coverage of all bacterial strains found in BSIs, coupled with the intrinsic limitation of CHROMagar Orientation to only identify a limited number of organisms. As a result, the main application of this protocol would be as a rapid screening method for the organisms presented in this work. As for the possibility to extend the screening for other strains covered by this specific CHROMagar, the strains tested exhibited a diverse range of sizes and shapes^50–52^, which are representative of the types of bacteria commonly found in clinical samples. Therefore, it can be inferred that the centrifugation methods employed in this study can be extrapolated to other types of bacteria, as the isolation efficiency of the centrifugation step relies mainly on physical properties (e.g. bacteria’s hydrodynamic radius). Moreover, although healthy donor blood may not fully replicate the physiological conditions of septic patient blood, its use in this study was necessary to establish technical feasibility under controlled settings. Future research with septic clinical blood samples is needed to validate these findings.

In summary, we demonstrate the direct isolation and identification of the most prevalent sepsis-causing bacteria from blood at clinically relevant concentrations using only standard hospital laboratory equipment, making it readily integrable in diagnostic pipelines of high, middle, and low-income countries. Our approach can be used for rapid, preliminary identification, and has the potential to be particularly valuable in low-resource settings or in situations where rapid presumptive ID can guide early management. With a sample-to-answer TAT of just 12 h, we believe this easily implementable protocol can enable the earlier administration of effective antibiotics and ultimately improve patient outcomes.

## Methods

### Bacterial strains

We used three commonly sepsis-causing bacteria, covering both Gram-negative and Gram-positive species. As Gram-positive representatives, we used *S. aureus* and *E. faecalis*, both clinical isolates from Karolinska Hospital (Stockholm, Sweden). As Gram-negative representatives, we used *E. coli* ATCC 11775, *K. pneumonia* ATCC 13883, both obtained from Uppsala University (Uppsala, Sweden), and *P. aeruginosa*, a clinical isolate from Karolinska Hospital (Stockholm, Sweden). Bacteria were long-term stored at $-80 ^{\circ }$C in standard glycerol solution.

### Preparation of spiked blood

Blood from anonymous healthy human donors (Blodcentralen, Stockholm, Sweden) was collected by a qualified phlebotomist directly into EDTA tubes and used for experiments no later than five days after collection. All the blood samples used were anonymous and obtained from healthy donors through a certified blood bank, solely for technical development purposes, and therefore no specific ethical approval was required under Swedish regulations. Informed consent was obtained from all human participants in this study.

To prepare for centrifugation, 2 mL of blood was pipetted inside a 15 mL falcon tube containing 4 mL of media from a standard aerobic hemocultucure bottle (BD BACTEC Plus Aerobic medium (BD, USA)), following the standard 1:2 (V/V) dilution performed clinically in hemocultures. The bacterial solution was prepared from the frozen glycerol stock, and grown to confluence overnight in BD BACTEC Plus Aerobic medium (BD, USA) at 37 $^{\circ }$C. The cultured bacterial solution was serially diluted to the desired concentration ($10^3$, $10^2$ or 10 CFU/mL) and used to spike the blood and media mixture. The exact concentration of the spiking solution was then determined by plating aliquots of the diluted solution, followed by colony counting on nutrient agar plates after overnight culture at 37 °C. During spiking, the bacterial solution volume was always less than 5% of the total volume.

### Bacteria quantitation

Agar plates were prepared by dissolving LB broth with agar (Miller) (Sigma-Aldrich, USA) in de-ionized water at 40 g/L concentration, followed by autoclaving and transferring on clean plates. A volume of 100 μL of the bacterial solutions from the spiking solution or isolated from the blood samples were plated in the agar plates. Bacteria quantitation was performed after plating the bacteria on the agar and overnight incubation at 37 $^{\circ }$C. The initial number of bacteria was calculated as the product of the whole blood bacterial concentration, $C_{b,blood}$, multiplied by the volume of blood ($V_{blood}$ = 2 mL). The number of bacteria recovered in the supernatant was calculated as the product of the supernatant bacterial concentration, $C_{b,SN}$, multiplied by the volume of supernatant recovered, ($V_{SN}$ = 4 mL). The bacteria isolation efficiency, *BI*, is then defined as the bacteria recovered in the supernatant divided by the initial bacteria in the whole blood.

$$\begin{aligned} BI = \dfrac{V_{SN}\cdot C_{b,SN}}{V_{blood}\cdot C_{b,blood}} = 2 \dfrac{C_{b,SN}}{C_{b,blood}} \end{aligned}$$

### Blood cell quantitation

The determination of the number of RBCs present in either whole blood or recovered plasma was achieved indirectly by assessing the level of hemoglobin within the sample. Indeed, hemoglobin shows an absorption peak at 540 nm^53^ that can be used in conjunction with the Beer-Lambert law to determine its concentration. Calibration experiments were performed, where a blood dilution ladder (in BCM) was loaded on a 96-well plate and ran through a photo spectrometer. The optical densities of these samples were measured at 540 nm wavelength. In parallel, undiluted blood was analyzed using a hemocytometer and the corresponding RBC count was retrieved. A calibration curve that linearly related the hemocytometer counts to the optical density values was generated (data not shown).

Unknown blood and plasma samples were then run through the spectrometer, and RBC counts were inferred from the 540 nm absorption using the aforementioned calibration curve. The red blood cell removal (RBCR) was defined as the percentage of RBC removed from the recovered supernatant with respect to the initial hemoculture. It is calculated analogously to the bacteria isolation efficiency BI:

$$\begin{aligned} RBCR = 1-\dfrac{N_{RBC,SN}}{N_{RBC,blood}} = 1-\dfrac{C_{RBC,SN}\cdot V_{SN}}{C_{RBC,blood}\cdot V_{blood}} = 1- 2 \dfrac{C_{RBC,SN}}{C_{RBC,blood}}, \end{aligned}$$

with $C_{RBC,SN}$ and $C_{RBC,blood}$ the RBC concentrations in the supernatant of the spun sample and the initial blood, respectively, and $N_{RBC,SN}$ and $N_{RBC,blood}$ the absolute numbers of RBC in the supernatant and the initial blood sample, respectively.

### Volume reduction

The supernatant obtained from the first bacterial isolation step (4 mL) was carefully removed by pipetting and placed in a 15 mL Falcon tube. The tube was centrifuged at 400*g* or 1000*g* for 10 min in a fixed rotor centrifuge (Clinispin CT20 Microcentrifuge, Woodley). For the volume reduction with density medium, a volume of 200 μL of Percoll (Sigma-Aldrich, USA), a density medium of density 1.125–1.135 g/ml was placed at the bottom of a 15 mL Falcon tube. The 4 mL of supernatant obtained from the single bacterial isolation step was layered on top of the density medium. The tube was centrifuged at 400*g* or 1000*g* for 10 min in a fixed rotor centrifuge (Clinispin CT20 Microcentrifuge, Woodley). In both cases, all excess liquid above 0.5–0.7 ml was removed without removing the pellet. The sample was then resuspended by vortexing.

### Chromogenic agar plates preparation

To perform species identification, we prepared plates of commercially available chromogenic agar. Plates were prepared by dissolving 33 g of powder base chromogenic agar, CHROMagar Orientation (CHROMagar, France) in 1 L of purified water. The mix was autoclaved for 15 min at $\hbox {121}^\circ$C, cooled down at room temperature for approximately 20 min, and poured into sterile Petri dish plates. The plates were then cooled down to room temperature until the agar mix had solidified and subsequently stored at $\hbox {2}^\circ$C for a maximum of two days.

### Colony imaging and image analysis

Images of the CHROMagar plates were taken using a commercial NIKON D5300 Camera, with a white background. The image analysis was performed on single cropped colonies in ImageJ software (ImageJ2 Version 2.14.0/1.54 m). The background of the images was subtracted and the boundaries of bacterial colonies were manually chosen as the areas of interest (SI Figure 2). For each colony, the average pixel intensity (in RGB space) was measured, yielding a triplet in a three-dimensional color space (R, G, B). Triplets can then be plotted to help identify distributions, trends or patterns in the dataset.

### Statistical analysis

Data analyses were performed with GraphPad Prism v10.0.0 (GraphPad Software, USA) for Windows. The data were analyzed under the assumptions of normality and homogeneity of variance. Statistically significant differences between treatments, bacterial species, and their interaction were assessed using a Two-Way ANOVA (Fischer’s LSD) or a student’s unpaired t-test. A *p*-value of < 0.05 was considered statistically significant.

## Supplementary Information

Supplementary Information.

## Data Availability

All data needed to evaluate the conclusions in the paper are present in the paper and/or the Supplementary Materials.
