# Supplementary material for: Isolation and identification of bacteria from blood within 12 h using standard laboratory equipment
Source: Sci Rep. 2025 Jul 9;15:24661. doi: 10.1038/s41598-025-09024-9 (PMC12241390; doi:10.1038/s41598-025-09024-9)
Supplement: Supplementary file 1 — Supplementary Information. [file 41598_2025_9024_MOESM1_ESM.pdf]

Supplementary information

Microbial Etiology of Sepsis

SI Table 1. Pathogen Prevalence in Sepsis: Summary of Studies.

**Source**	Komori et. al[3]	Tabah et. al[35]	Umemura et. al[36]	Opota et. al[6]
**Geography**	Japan	Worldwide	Japan	Switzerland
**Patient Type**	ICU: sepsis + bacteremia	ICU: hospital-acquired BSIs	ICU: sepsis	Bacteremia
***E. coli* (%)**	39.0	7.4	21.5	28.6
***K. pneumoniae* (%)**	11.7	11.9	9.0	7.1
***S. aureus* (%)**	22.1	9.0	9.5	13.6
***E. faecalis* (%)**	2.5	10.9	4.3	< 5
***P. aeruginosa* (%)**	2.0	11.4	4.1	8.5
**Cumulative Sum (%)**	77.3	50.6	48.4	> 49.3

SI Table 2. Statistical description of RGB intensity for the analyzed bacterial colonies of *E. coli* (n=18), *K. pneumoniae* (n=18), *S. aureus* (n=25), *E. faecalis* (n=9) and *P. aeruginosa* (n=21). Mean and standard deviation are described.

	E. coli	*K. pneumoniae*	*E. faecalis*	*S. aureus*	*P. aeruginosa*
Red	241±2	171±3	209±10	251±1	252±0.4
Green	223±3	246±2	241±4	249±1	251±1
Blue	246±2	235±3	249±3	238±3	246±1

Optimization of the operational centrifugal parameters for isolation of bacteria from hemocultures

This protocol makes use of the velocity differential between RBCs and bacteria during sedimentation. The former is estimated to be around thirty times faster than the latter[37]. This can be taken advantage of by spinning the culture blood long and hard enough so that the larger RBCs sediment at the bottom of the tube, but slow and short enough so that the bacteria are still contained within the clearer plasma fraction. To compare experiments with different spin profiles, we measured the RBCs removal, and the bacteria isolation efficiency for each parameter. The choice of 500g for 2 min has been chosen empirically by maximizing the recovery of *E. coli* and red blood cell (RBC) removal. These values, along with those from the other centrifugal settings tested can be seen in SI Figure1.

As spin time and acceleration increase, the bacteria isolation rate goes down, but red blood cell removal goes up. We found that for 2 mL spiked whole blood mixed with 4 mL BCM (the standard 1:2 dilution mixture obtained during patient sampling) in a 15 mL falcon tube, centrifuging at 500g for 2 min was long enough for 99.36 +/- 0.35% blood cell removal, while short enough to recover 55.71 +/- 4.92% bacteria, which we chose as an optimal middle-ground.

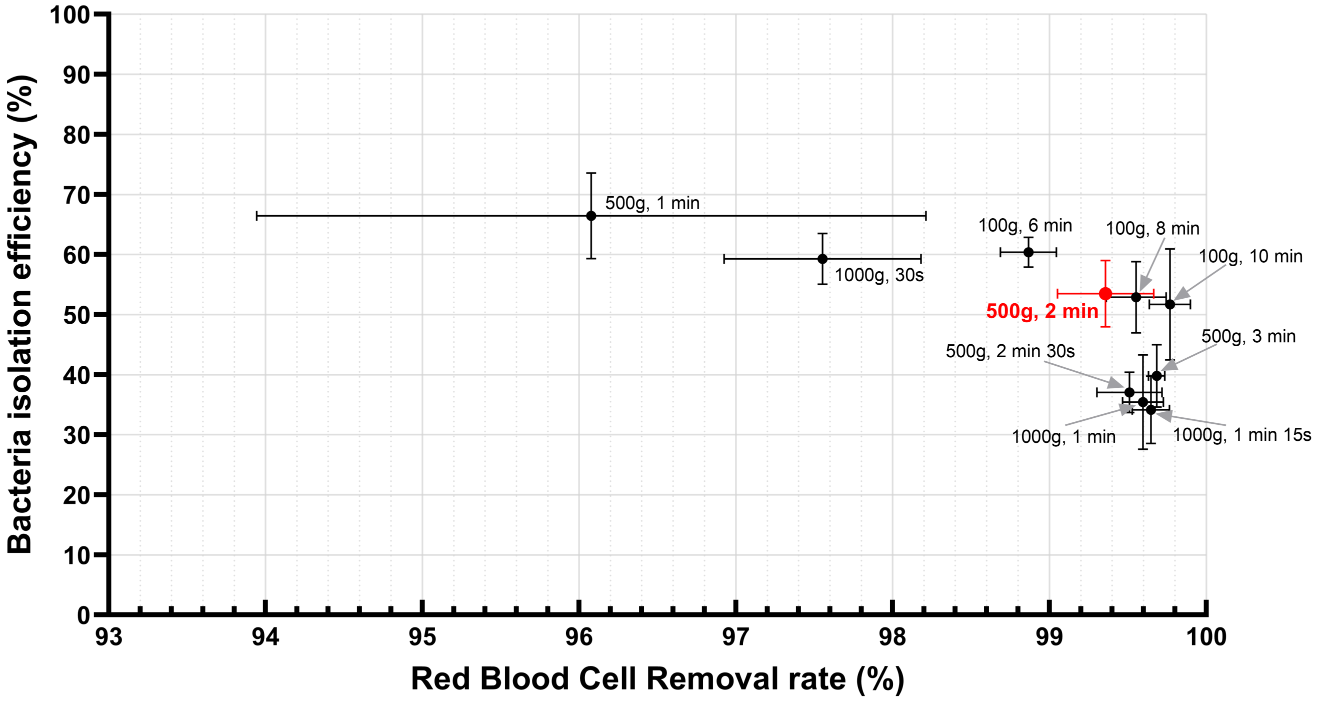

SI Figure 1. *E. coli* bacteria isolation efficiency after first centrifugation step graphed against RBC removal at different centrifugation parameters. For each set of parameters, n=4 experiments were performed. Error bars show standard deviation.

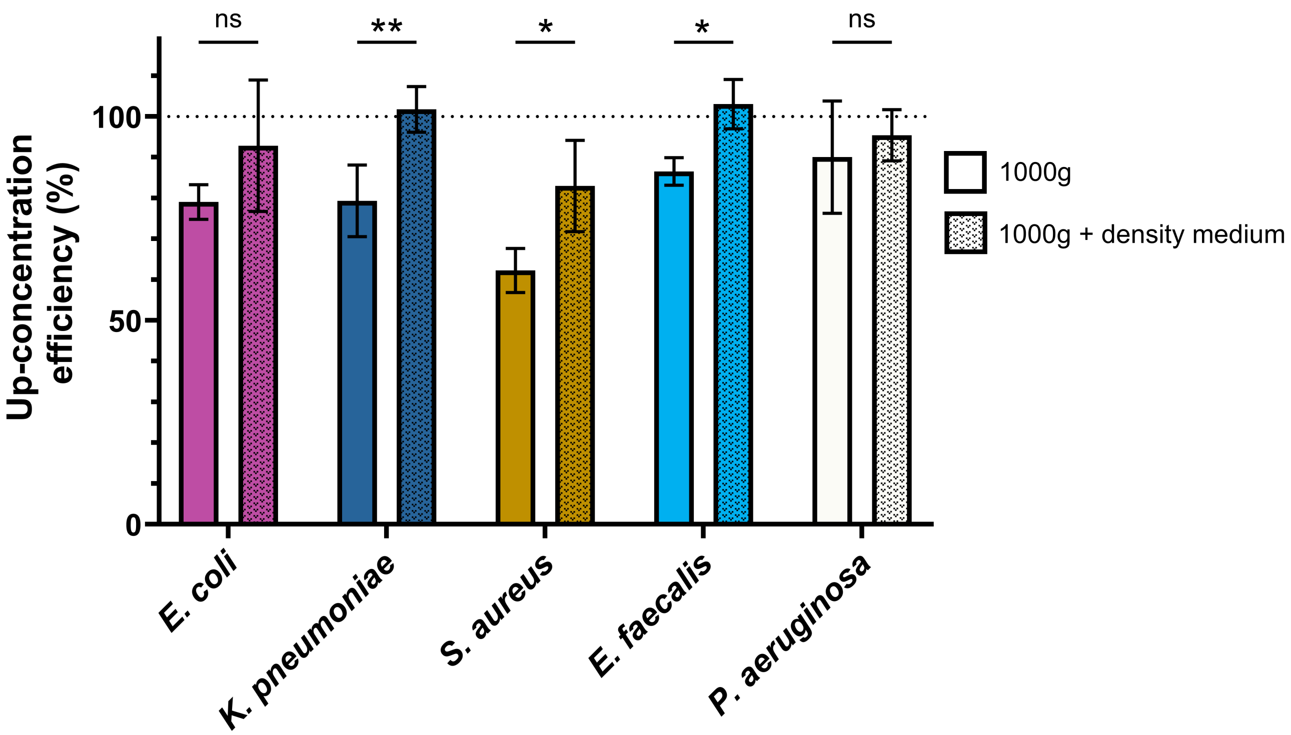

SI Figure 2. Efficiency of pelleting step (percentage of bacteria recovered after pelleting compared to the bacterial count before pelleting) at 1000g with and without density medium. Data for all five bacterial species are shown, with mean and standard deviation. Statistical test: Unpaired two-tailed t-tests; ns indicates significance level with p-value ≥ 0.05, * indicates significance level with p-value < 0.05 and ** indicates significance level with p-value < 0.01.

Image Analysis of ChromAgar Bacterial Colonies

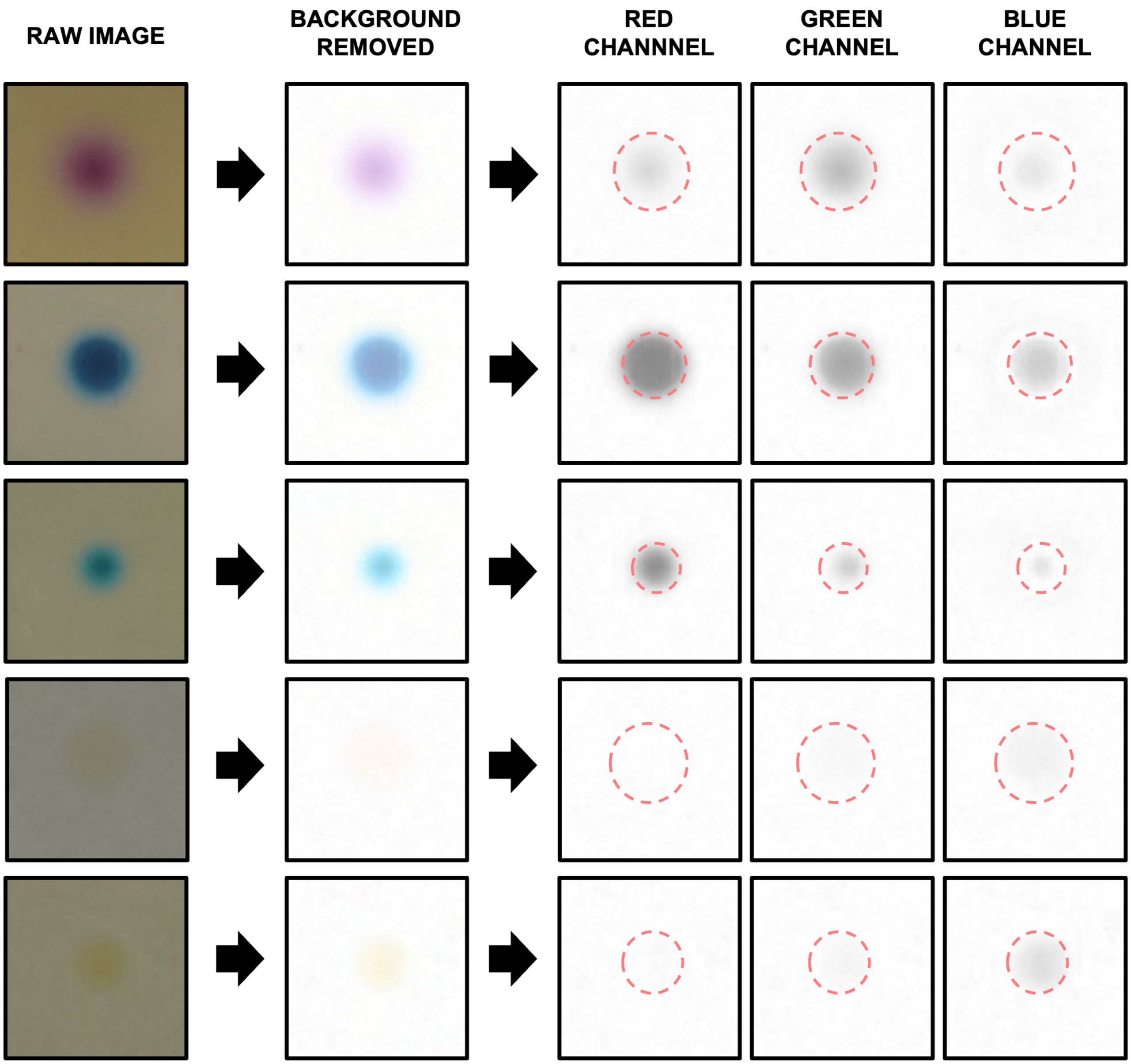

SI Figure 3. Image analysis pipeline of ChromAgar bacterial colonies for *E. coli*, *K. pneumoniae*, *E. faecalis*, *P. aeruginosa* and *S. aureus* (respectively, from top to bottom).

To analyze the single colony images, images were uploaded to Fiji (ImageJ), where the background of the image was subtracted with the command Process> Subtract background. The pixel radius was selected to be 100 pixels. The different color channels were split (Image>Color>Split Channel), and a ROI (region of interest) was defined around the colony. Average pixel intensity of the ROI was measured, recorded and saved onto a .csv file for each of the color channels. The overall image processing pipeline is shown in SI Figure 3. The data obtained from these measurements was used for plotting the values of the individual colonies in Figure 4B. The values presented in SI Table 2 recapitulate the RGB intensities obtained for each bacterial strain cluster after the aforementioned image processing.
